# Spectrum of Cytogenetic and Molecular Abnormalities in Disorders of Sex Development: A Retrospective Observational Study From Western India

**DOI:** 10.7759/cureus.110941

**Published:** 2026-06-16

**Authors:** Suchi Acharya, Barun Chakrabarty, Naresh Bansal, Shuvendu Roy, Narendra Kotwal, Vikas Dagar

**Affiliations:** 1 Pediatrics, Armed Forces Medical College, Pune, IND; 2 Pathology and Laboratory Medicine, Armed Forces Medical College, Pune, IND; 3 Endocrinology and Metabolism, Armed Forces Medical College, Pune, IND; 4 Endocrinology, Paras Hospital, Panchkula, IND; 5 Pathology, All India Institute of Medical Sciences, Kalyani, Kalyani, IND

**Keywords:** dihydrotestosterone, disorders of sex development, karyotype, testosterone (tt), whole exome sequence

## Abstract

Background: Disorders of sex development (DSD) are a social and medical emergency. A thorough history, detailed clinical examination, and appropriate investigations will help to identify the aetiology of DSD. But still, in most cases, the exact aetiology is not reached. Reaching a definite and firm diagnosis with the help of genetic testing is vital for the long-term management of DSD patients.

Methods: We conducted a hospital record-based, retrospective cross-sectional study of 20 children with DSD aged 0-14 years who attended the paediatric endocrine clinic at our institute from February 2022 to July 2024. Clinical, hormonal, radiological, cytogenetic, molecular and outcome data were analysed.

Results: During the study period, 20 children were diagnosed with DSD at our institute. Their age range was between 0 and 14 years. Among them, 6/20 patients were diagnosed with 46, XX DSD, 12/20 with 46, XY DSD and 2/20 with sex chromosomal DSD. Cytogenetic analysis and whole-exome sequencing were done in all patients. Seven patients (35%) showed significant pathogenic variants in genes such as RSPO1, CYP21A2, CYP19A1, WT1, and SRD5A2. Out of seven genetically confirmed DSD patients, in only one was the gender of rearing changed after the diagnosis.

Conclusion: Management of DSD involves a multidisciplinary team from different specialities. The present study focuses on the cytogenetic and molecular diagnostic findings in children with atypical genitalia, which can be an efficient tool in the clinical diagnosis and management of DSD.

## Introduction

Discordance between chromosomal, gonadal and anatomical sex is called a disorder of sex development (DSD) [1]. Its incidence is 1 in 1000 to 4500 live births (exact data is limited) [2,3]. DSD is broadly classified into four major categories: 46, XX DSD, 46, XY DSD, sex chromosome DSD and ovotesticular DSD [1]. Normal human sexual development is tightly controlled by genetic, environmental, and hormonal factors, especially androgens. There are several genes involved in the differentiation and development of gonads, like SRY, NR5A1, DAX1, SOX9 and WT1, which are responsible for testicular development and differentiation, while R-spondin 1 (Rspo1)/Wnt-4/beta-catenin signalling genes help in ovarian differentiation and development [4-6].

In most cases of DSD, the exact aetiology is not reached even after applying a battery of tests. Genetics and cytogenetics help confirm a diagnosis in such cases. Knowing the exact aetiology of DSD is important as it will help in patient management in relation to possible gender assignment, assessment of adrenal and gonadal functions, future risk of gonadal cancer, recurrence in future pregnancies and long-term outcomes associated with it. Several genetic tools, such as fluorescence in situ hybridisation (FISH), microarrays, and next-generation sequencing (NGS), have ushered in a new era of definitive diagnosis in children with DSD [7]. The present study focuses on the cytogenetic and molecular diagnostic findings in children with atypical genitalia, which can be an efficient tool in the clinical diagnosis and management of DSD.

## Materials and methods

Study design and setting

A retrospective cross-sectional study was carried out in a western Indian tertiary care teaching institution's paediatric endocrinology outpatient department. We retrieved and examined hospital-based records of children aged up to 14 years diagnosed with DSD identified between February 2022 and July 2024. The objective of the study was to characterise cytogenetic and molecular abnormalities in children with atypical genitalia undergoing evaluation for DSD.

Study population

Children between the ages of 0 and 14 who satisfied the diagnostic criteria for DSD [1] and visited the Paediatric Endocrinology Clinic during the study period were included. Patient confidentiality and anonymity were strictly maintained, and written informed consent/assent for publication of clinical photographs was obtained from either of the parents/legal guardians. Analysis did not include patients with incomplete records or who lacked necessary laboratory, clinical, cytogenetic, or molecular genetic tests.

Inclusion and exclusion criteria

Children presenting with micropenis, hypoplastic or bifid scrotum, bilateral cryptorchidism, unilateral cryptorchidism with hypospadias, penoscrotal or perineoscrotal hypospadias, clitoromegaly, or any overt genital anomalies such as cloacal exstrophy or asymmetry of labioscrotal folds were included. All patients were classified as having DSD according to the Chicago Consensus Classification, which categorises DSD into sex chromosome DSD, 46,XY DSD, and 46,XX DSD. This classification was first proposed in 2006 and subsequently reaffirmed in the 2016 Global DSD Update [1,3]. Patients with incomplete clinical, biochemical, radiological, cytogenetic, or molecular data and those without a confirmed diagnosis of DSD were excluded. Cases with insufficient records for definitive classification were also excluded.

Data collection

Data were extracted from medical records using a structured data collection proforma. Recorded variables included socio-demographic information (age, gender of rearing, place of residence), clinical parameters (blood pressure, anthropometry, stretched penile length (SPL), clitoral length, presence and location of palpable gonads, scrotal symmetry, development of labioscrotal folds, and degree of genital pigmentation). Standardised scoring systems were used to characterise genital ambiguity, including the External Masculinization Score (EMS), Prader staging for virilisation in individuals with a predominantly female phenotype, and Quigley staging for androgen insensitivity syndromes (AIS). Micropenis was defined as an SPL less than -2.5 standard deviations below the age-specific mean [8]. An EMS score below 11 was considered indicative of genital ambiguity requiring detailed evaluation [2]. All patients underwent comprehensive hormonal and biochemical assessment as part of the diagnostic workup. Hormonal investigations included luteinizing hormone (LH), follicle-stimulating hormone (FSH), testosterone (basal and post-human chorionic gonadotropin (hCG) stimulation - a standard paediatric three-day hCG stimulation protocol was performed using age-adjusted doses (500 IU/day for <1 year, 1,000 IU/day for 1-10 years, and 1,500 IU/day for >10 years), followed by measurement of serum testosterone and dihydrotestosterone (DHT) levels where indicated), DHT, androstenedione, dehydroepiandrosterone (DHEA), 17-hydroxyprogesterone (17-OHP), cortisol, estradiol, adrenocorticotropic hormone (ACTH), and thyroid-stimulating hormone (TSH). Serum electrolytes were evaluated to identify salt-wasting states and other metabolic abnormalities. Hormonal assays were performed using chemiluminescent microparticle immunoassay (CMIA), and 17-OHP levels were measured using enzyme-linked immunosorbent assay (ELISA). All patients underwent radiological imaging, including abdominal and pelvic ultrasonography (USG) and magnetic resonance imaging (MRI) of the pelvis. Diagnostic laparoscopy, exploratory laparotomy, and gonadal biopsy were performed when clinically indicated. Surgical findings, gonadal morphology, and histopathological examination results were recorded and correlated with clinical and genetic findings.

Cytogenomic analysis

Karyotypic Analysis

Conventional karyotyping was performed on 2-3mL of peripheral blood collected in a sodium heparin vacutainer using standard short-term lymphocyte culture techniques, and G-banded karyotyping was performed. Standard short-term culture procedures were followed to establish peripheral blood lymphocyte cultures, and G-banded karyotyping was performed. For each patient, at least 20 metaphase spreads were examined. To increase detection sensitivity in situations where chromosomal mosaicism was suspected, analysis was expanded to at least 50 metaphases.

Molecular Cytogenetic Analysis

All patients underwent FISH utilising centromeric enumeration probes for chromosomes X and Y (CEPX/Y; MetaSystems, Germany). For interpretation, 200 interphase non-overlapping cell signals were evaluated, and the reporting was done as per the International System of Cytogenomic Nomenclature 2024 guideline. The FISH study confirmed karyotype results, detected low-level mosaicism, and verified the chromosomal constitution.

Molecular Genetic Analysis

For molecular characterisation, genomic DNA was extracted from 1-2 mL ethylenediaminetetraacetic acid (EDTA)-anticoagulated peripheral blood using the QIAamp DNA Blood Mini Kit (QIAGEN GmbH, Hilden, Germany). Whole-exome sequencing (WES) was performed using the Illumina NextSeq 2000 platform (Illumina, Inc., San Diego, CA, USA) using the Clinical Exome Version 6 platform, covering approximately 7,000 clinically relevant genes curated from the Online Mendelian Inheritance in Man (OMIM), ClinVar, and Human Gene Mutation Database (HGMD) databases. The assay provides comprehensive coverage of all protein-coding regions (exons) across approximately 23,000 genes, including genes implicated in gonadal development, sex determination, sex steroid biosynthesis, and hormone action. Sequencing achieved high analytical sensitivity with a mean coverage depth exceeding 80-100× across clinically relevant DSD-associated genes.

Bioinformatic analysis was performed using the VarMiner pipeline. Variant calling included single nucleotide variants (SNVs), insertions/deletions (InDels), and copy number variants (CNVs). Targeted filtering focused on established DSD-associated genes, including but not limited to SRY, SOX9, NR5A1 (SF1), WT1, MAP3K1, AR, CYP17A1, HSD17B3, ARX, and other genes implicated in sex development disorders. Variant interpretation followed Association for Molecular Pathology (AMP) and the American College of Medical Genetics and Genomics (ACMG) guidelines wherever applicable [9].

Multiplex ligation-dependent probe amplification (MLPA) was performed in selected cases where copy number abnormalities were suspected or where sequencing findings warranted confirmation of exon-level deletions or duplications. Due to limitations in sample availability and the retrospective nature of the study, Sanger sequencing validation and parental segregation analysis could not be performed in all cases and have been acknowledged as study limitations.

Variants classified as pathogenic or likely pathogenic and showing concordance with the clinical phenotype were considered diagnostic. Variants of uncertain significance (VUS) were interpreted cautiously and were not considered definitive unless supported by strong phenotypic and literature evidence. Cases with negative molecular findings were retained as genetically unresolved and were interpreted in conjunction with clinical, hormonal, imaging, cytogenetic, and FISH findings.

Outcome measures

**Figure 1:**
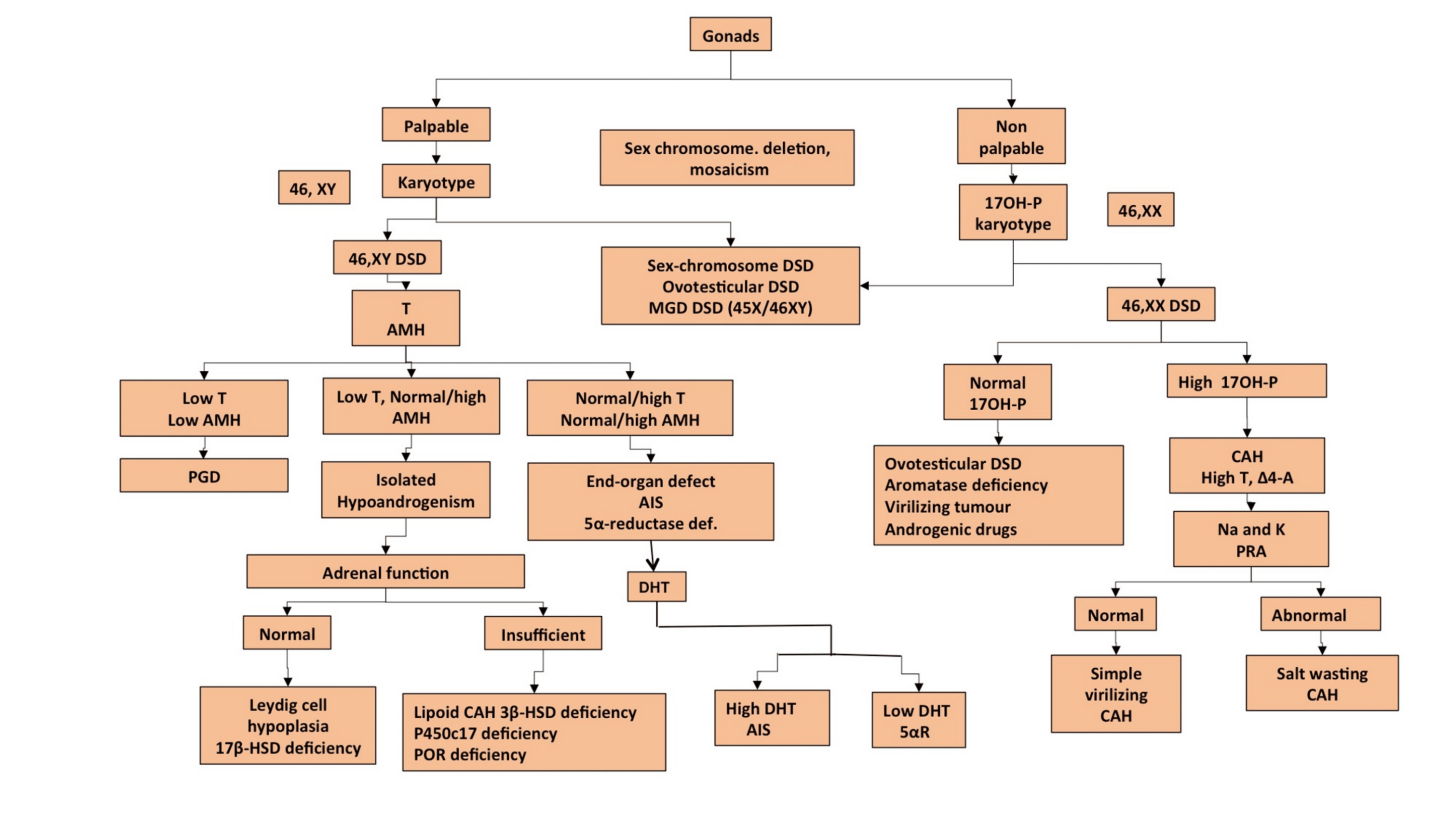
Approach to ambiguous genitalia in children AMH: anti-Müllerian hormone; CAH: congenital adrenal hyperplasia; DSD: differences of sex development; T: testosterone; 17-OHP: 17-hydroxyprogesterone; 17β-HSD: 17β-hydroxysteroid dehydrogenase; 3β-HSD: 3β-hydroxysteroid dehydrogenase; POR deficiency: cytochrome P450 oxidoreductase deficiency; DHT: dihydrotestosterone; AIS: androgen insensitivity syndrome; 5αR: 5-alpha-reductase type 2 deficiency; Na: sodium; K: potassium; PRA: plasma renin activity and aldosterone; MGD: mixed gonadal dysgenesis; PGD: pure gonadal dysgenesis

Patients were evaluated using the standardised DSD diagnostic algorithm (Figure 1) and classified as 46, XX DSD, 46, XY DSD, or sex chromosome DSD. All cases underwent karyotyping and WES, with variants classified according to ACMG guidelines; MLPA was performed where indicated. Cases without pathogenic, likely pathogenic, or clinically relevant variants were further assessed clinically.

Establishing a possible aetiological diagnosis with combined clinical, biochemical, radiological, cytogenetic, and molecular genetic examination was the primary outcome. Secondary outcomes included medical interventions, surgical procedures, management choices depending on the final diagnosis, and changes in gender assignment or reassignment following a conclusive diagnosis.

Statistical analysis

A secure database was used to store the data, and the relevant statistical software was used for analysis. Depending on the distribution of the data, continuous variables were summarised as mean ± standard deviation or median with interquartile range. Frequencies and percentages were used to express categorical variables. The study cohort's clinical spectrum, genetic causes, and consequences of DSD were described using descriptive analysis.

## Results

**Figure 2:**
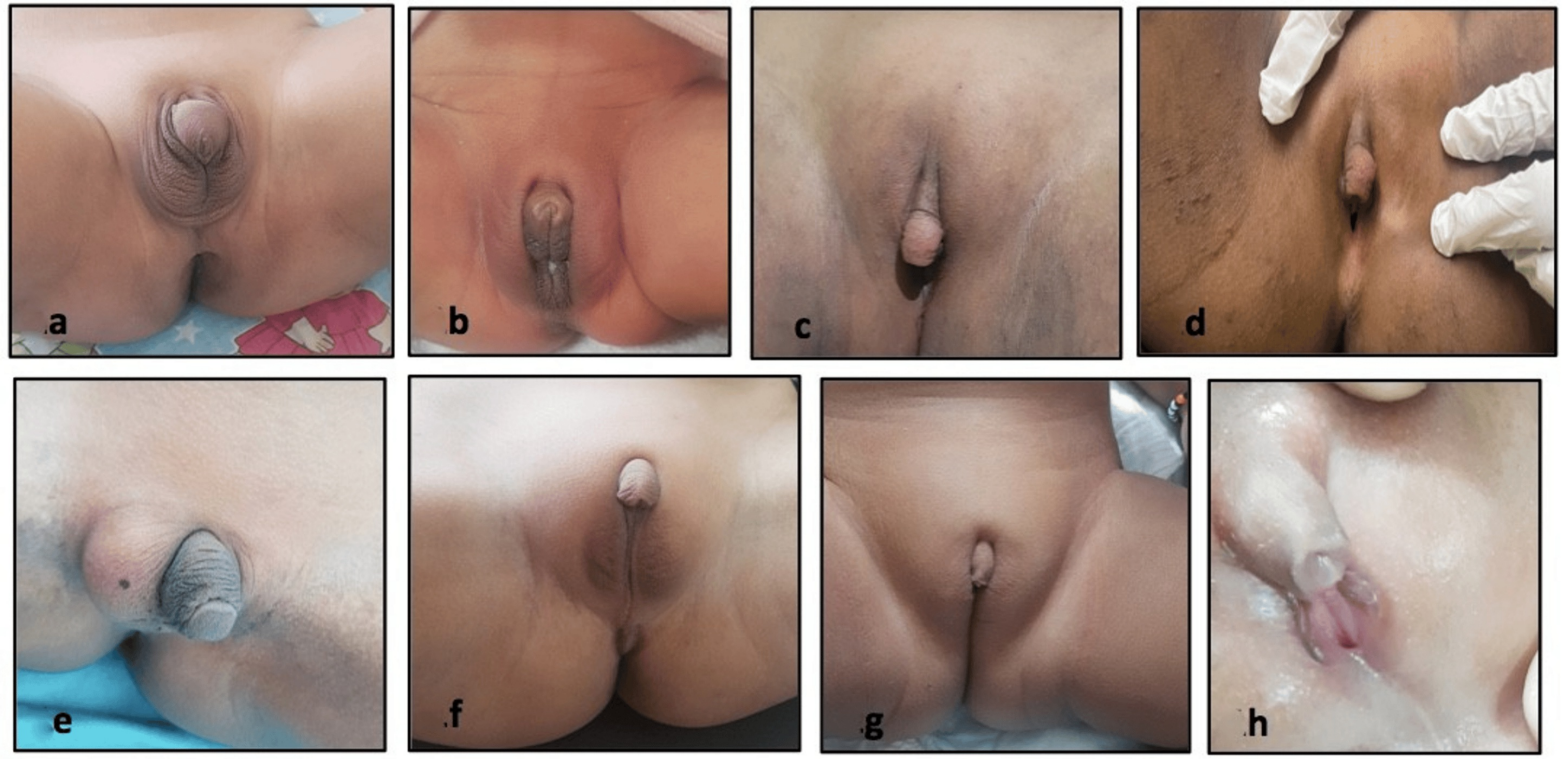
Genital finding in different cases (a) Genital findings in 46,XX testicular DSD showing micropenis and normal scrotal folds. (b) Genital findings in congenital adrenal hyperplasia (CAH) showing clitoromegaly and hyperpigmentation. (c, d) Genital findings in aromatase deficiency showing clitoromegaly and a vaginal opening. (e) Genital findings in 45,X/47,XYY DSD showing micropenis, an asymmetrical scrotum, and a left undescended testis. (f) Genital findings in Denys-Drash syndrome showing micropenis and a hypoplastic scrotum with bilateral undescended testes. (g) Genital findings in 5-alpha-reductase type 2 deficiency showing micropenis, a bifid scrotum, and bilateral undescended testes. (h) Vaginal opening in a patient with 5-alpha-reductase type 2 deficiency. DSD: differences of sex development

**Figure 3:**
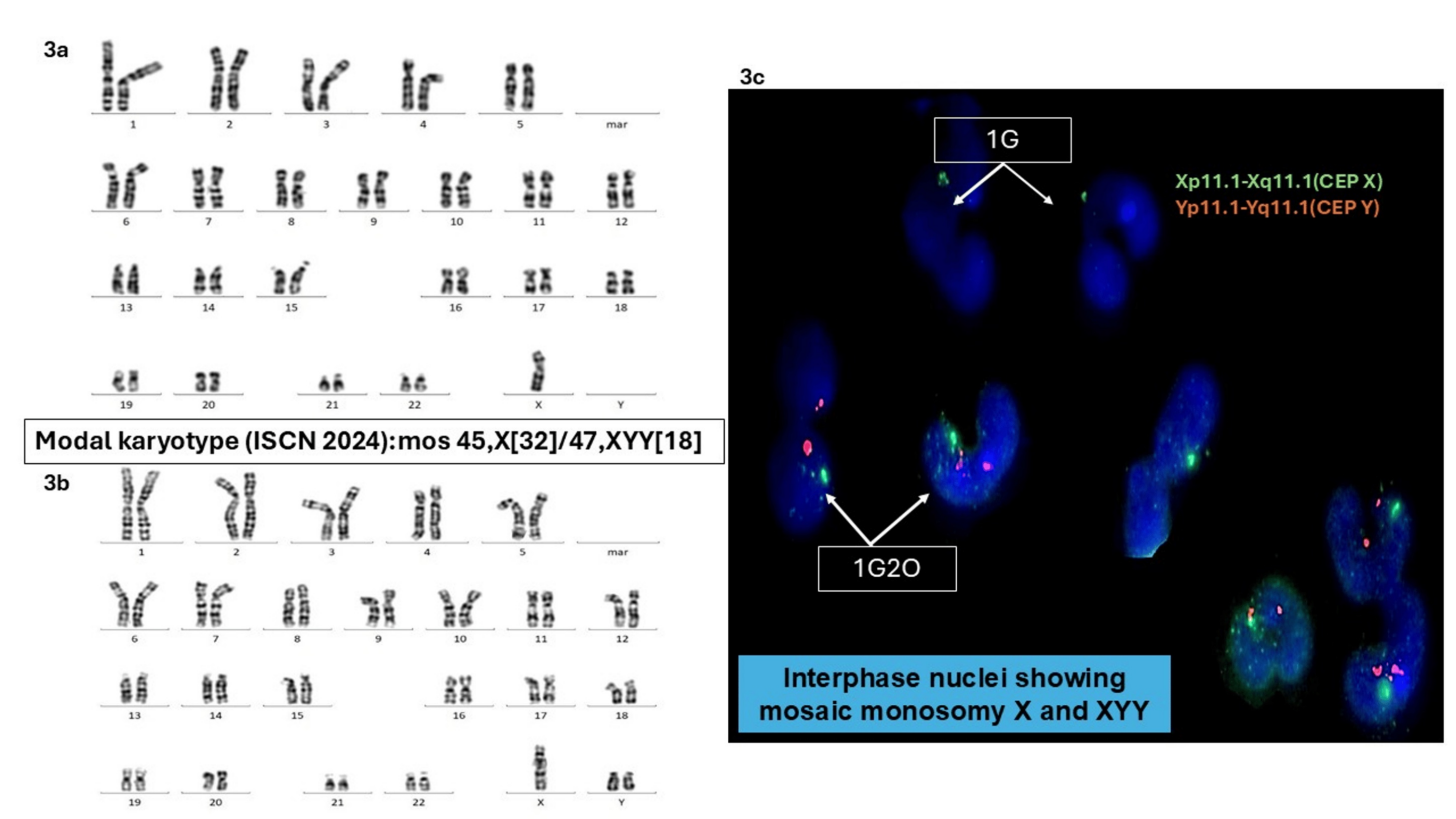
Karyotype and fluorescence in situ hybridisation finding in Case 4 (a, b) Karyograms showing mosaic 45,X/47,XYY. (c) FISH analysis using a centromeric XY probe on interphase cells showing the presence of mosaicism, with some cells exhibiting a single green hybridisation signal (1G) for the X chromosome admixed with cells exhibiting one green hybridisation signal for the X chromosome and two orange hybridisation signals (1G2O) for the Y chromosome. The 1G pattern (white arrow pointing downward) indicates a single green signal corresponding to the X chromosome with no orange signal, whereas the 1G2O pattern (white arrow pointing upward) indicates one green signal for the X chromosome and two orange signals for the Y chromosome.

Twenty DSD patients presented at our institute during the study period. Most of them (95%) lived in urban areas. Age at presentation ranged from one month to 14 years. The majority (62%) presented before the age of 10 (Table 1). Of 20 DSD patients, 6/20 (30%) were 46, XX DSD, 12/20 (60%) were 46, XY DSD, and 2/20 (10%) were sex chromosomal DSD (Table 1). In 46, XX DSD, two out of six were diagnosed as congenital adrenal hyperplasia (CAH), two out of six were testicular DSD, and one each was aromatase deficiency and ovotesticular DSD. In 46XY DSD, 5/12 had AIS, 2/12 had partial gonadal dysgenesis (gonadal biopsy showed a dysgenetic testis), 2/12 had 5-alpha-reductase type 2 deficiency, 1/12 had CAH, and 1/12 had Denys-Drash or ovotesticular DSD. There were two cases of sex chromosomal DSD presented as mixed gonadal dysgenesis (MGD). The chromosomal karyotypes were mosaic 45, XO/46, XY and 45, XO/47, XYY, respectively. One patient, belonging to 46, XY DSD (5 alpha reductase type 2 deficiency), underwent gender reassignment. The detailed clinical, biochemical, and genetic profiles of these children are presented in Table 1 and Figures 2-3. Of 20 DSD children, seven (35%) carried a pathogenic variant in RSPO1, CYP21A2, CYP19A1, WT1, or SRD5A2. One sex chromosomal DSD underwent FISH and found a very rare finding of mosaic 45, XO/47, XYY. The remaining 13 cases showed normal karyotypes and no pathogenic or likely pathogenic variants on WES. These cases were classified as genetically unresolved; however, they were categorised according to the diagnostic algorithm (Figure 1) based on their clinical, hormonal, and biochemical findings. Parental segregation analysis was advised where appropriate but could not be performed due to financial constraints. Hence, these patients were categorised and managed based on the best available clinical, biochemical, radiological, and genetic information. The detailed case discussion of all these genetically diagnosed DSD patients is given below.

**Table 1:** Detailed clinical, hormonal, and genetic profile of DSD children (N=20) 5ARD2: 5-alpha-reductase type 2 deficiency; AD: aromatase deficiency; AMH: anti-Müllerian hormone; B/L: bilateral; CAH: congenital adrenal hyperplasia; DDS: Denys-Drash syndrome; DHT: dihydrotestosterone; DSD: differences of sex development; G: gonads; MGD: mixed gonadal dysgenesis; MS: Müllerian structures; NA: not applicable; N: normal; 17-OHP: 17-hydroxyprogesterone; OVT DSD: ovotesticular DSD; PGD: partial gonadal dysgenesis; T: testosterone; T-DSD: testicular DSD; U/L: unilateral; WES: whole-exome sequencing

Parameters	46, XX DSD (n=6)	46, XY DSD (n=12)	Sex Chromosomal DSD (n=2)	Total (n=20)
Age				
<1 years	2	4	0	6 (30%)
1-5 years	3	5	1	9 (45%)
>5 years	1	3	1	5 (25%)
Gender of rearing				
M	2	10	2	14 (70%)
F	4	2	0	6 (30%)
Gender reassignment	0	1	0	1 (5%)
Consanguinity	2	1	0	3 (15%)
Rural	0	1	0	1 (5%)
Urban	6	11	2	19 (95%)
Family H/O DSD	2	0	0	2 (10%)
Prader stage				
1	1	NA	NA	-
2	2	NA	NA	-
3	1	NA	NA	-
4	2	NA	NA	-
Hypospadias	2	9	2	13
Microphallus	2	11	2	15
Clitoromegaly	3	0	0	3
Vaginal opening	5	5	0	10
Gonads				
B/L palpable	1	7 (inguinal region-4, scrotum-3)	0	-
U/L palpable	0	2	2	-
NP	5	3	0	-
Testis	2 (testicular DSD)	11	1	-
Ovary	3	0	1	-
Ovotestis	1	1	0	-
Dysgenetic/streak testis	1 (testicular DSD)	2 (PGD)	1	-
Mullerian structures	4	2	1 (fallopian tubes in 45 X/47XYY)	-
High 17-OHP	2	1	0	-
Testosterone				
High	1	5	0	-
Low	5	7	2	-
T/DHT ratio (>17)	NA	NA	2 (5ARD2)	-
AMH				
Normal	1	9	1	-
Low	NA	3	1	-
WES				
RSPO-1	2	NA	NA	-
CYP21A2	2	NA	NA	-
CYP19A1	1	NA	NA	-
WT1	NA	1	NA	-
SRD5A2	NA	1	NA	-
Final diagnosis	CAH-2, Testicular DSD-2, AD-1, OVT DSD-1	AIS-5, PGD-2, 5ARD2-2, CAH-1, DDS-1, OVT DSD-1	MGD-2	-

Case 1

A five-year-old, developmentally normal child reared as a boy presented with microphallus, a small scrotum, and penoscrotal hypospadias (Figure 2a). He also had palmoplantar hyperkeratosis, squint, microcornea and microophthalmia of the left eye. The detailed clinical examination is described in Table 2. His younger sibling, aged 3.2 years, also had atypical genitalia. Investigations showed a 46, XX karyotype. FISH analysis showed the absence of the SRY gene. Biochemical evaluation showed normal LH, FSH, and AMH for age, with low testosterone and DHT (Table 3). MRI abdomen identified bilateral testes in the scrotal sac and absence of any mullerian structure (Table 4). Bilateral gonadal biopsy revealed testicular tissues on histopathology. WES reported a homozygous single-base pair deletion in exon 4 of the RSPO1 gene (Table 4). He was diagnosed as 46, XX testicular DSD with palmoplantar hyperkeratosis. Parents were counselled regarding the rearing of this child, hormone replacement therapy and appropriate surgical interventions. Parents opted for male rearing.

**Table 2:** Detailed clinical-epidemiological profile of genetically diagnosed DSD cases CL: clitoral length; CKD: chronic kidney disease; DSD: disorders of sex development; EMS: External Masculinization Score (*EMS <11 was considered indicative of DSD); FSGS: focal segmental glomerulosclerosis; h/o: history of; ID: intellectual disability; L: left; LE: left eye; NA: not available; NP: not palpable; p: percentile; P: palpable; R: right; SPL: stretched penile length

S. No.	Case 1	Case 2	Case 3	Case 4	Case 5	Case 6	Case 7
Karyotype	46, XX	46, XX	46, XX	45,XO/47,XYY	46, XY	46, XY	46, XX
Age	5 years	1 month	12 years	10.5 years	6 years	9 months	1 month
Height (cm)	106 (3^rd^ -50^th^ p)	51 (3^rd^ -50^th^ p)	139 (10^th^-25^th^ p)	129 (10^th^-25^th^ p)	110 (10^th^-25^th^ p)	69 (3^rd^ -50^th^ p)	50 (3^rd^ -50^th^ p)
SMR	NA	NA	1	1	1	NA	NA
Rural/urban	Urban	Urban	Urban	Urban	Urban	Rural	Urban
Gender of rearing	Male	Female	Female	Male	Male	Female	Female
Gender reassignment	No	No	No	No	No	Yes, male	No
Consanguinity	Absent	Absent	Present	Absent	Absent	Present	Absent
Family h/o DSD/others	Present	Absent	Absent	Absent	Father-CKD	Absent	Absent
SPL (cm)	3.0	NA	NA	3.8	3.0	1.7	NA
Bilateral gonads	b/l palpable in scrotum	R-NP L-NP	R-NP L-NP	R-P L- NP	R- inguinal L- inguinal	R- inguinal L- inguinal	NP
Scrotum	Small	NA	NA	Asymmetric, hypoplastic	Hypoplastic, poor rugosity	Symmetric, bifid	NA
Urethral opening	Penoscrotal hypospadias	Present	Present	Penoscrotal hypospadias	Penoscrotal hypospadias	Penoscrotal hypospadias	Present
Pigmentation	Normal	Dark	Normal	Normal	Normal	Normal	Dark
Anal opening	Present	Present	Present	Present	Present	Present	Present
CL (cm)	NA	1.8	2.5	NA	NA	NA	1.7
Posterior labial fusion	NA	Absent	Absent	NA	NA	NA	Absent
Separated labia	NA	Yes	Yes	NA	NA	NA	Yes
Vaginal opening	Absent	Present	Present	Absent	Absent	Present	Present
*EMS (12)	6	NA	NA	3.5	4	3	NA
Prader satge	4	2	1	NA	NA	NA	2
Quigley stage	2	NA	NA	1	3	4	NA
Dysmorphism/others	LE-squint, microcorneamicroopthalmia, palmoplantar keratosis	Nil	Nil	Primary hypothyroidism	Nephrotic syndrome (FSGS)	Nil	Nil

**Table 3:** Detailed laboratory investigations of genetically diagnosed DSD cases AMH: anti-Müllerian hormone; b/l: bilateral; CAH: congenital adrenal hyperplasia; CKD: chronic kidney disease; Cr: creatinine; DHT: dihydrotestosterone; DHEA: dehydroepiandrosterone; DSD: differences of sex development; FSH: follicle-stimulating hormone; G: gonads; GH: growth hormone; Hb: hemoglobin; hCG: human chorionic gonadotropin; HRT: hormone replacement therapy; K: potassium; L: left; LH: luteinizing hormone; MGD: mixed gonadal dysgenesis; MS: Müllerian structures; NA: not applicable; Na: sodium; N: normal; NS: nephrotic syndrome; NV: not visualized; 17-OHP: 17-hydroxyprogesterone; Plt: platelet count; Pre: pre-hCG stimulation; Post: post-hCG stimulation; RBS: random blood sugar; R: right; T: testosterone; T/A ratio: testosterone/androstenedione ratio; TSH: thyroid-stimulating hormone

S. No.	Case 1	Case 2	Case 3	Case 4	Case 5	Case 6	Case 7
Karyotype	46, XX	46, XX	46, XX	45,XO/47,XYY	46, XY	46, XY	46, XX
Hb, g/dL	10.1	14.2	11.1	10.5	11.3	11.9	13.2
Plt, L/cmm	3.5	3.2	4.2	3.7	2.7	3.8	1.7
Na, meq/L	138	131	139	136.5	140	138.2	128
K, meq/L	4.2	5.9	4.2	3,9	4.2	4.5	5.2
RBS, mg/dL	120	30	136	141	142	98	40
Cr, mg/dL	0.6	0.3	0.7	0.7	0.5	0.7	0.3
TSH (uIU/mL)	1.34	4.1	2.8	20.8	2.5	3.6	4.5
17 OHP, ng/mL (0.07-1.7)	0.08	63.5	0.04	0.03	0.04	0.05	78.5
Cortisol, mcg/dL (3-21)	9.58	0.2	8.6	7.5	8.2	7.2	0.25
ACTH, pg/mL (15-65)	NA	1250	28	NA	NA	32	1155
LH, IU/L (0.5-10)	0.12	0.13	9	0.4	0.61	0.12	0.13
FSH, IU/L (1.3-11.5)	0.13	0.9	28	0.3	1.0	0.3	0.3
T, ng/mL (2.2-10.3)	Pre-0.08 Post-0.12	Pre- 2.5 Post-NA	Pre-21 Post-NA	Pre-0.18 Post-0.24	Pre-0.19 Post-0.23	Pre-20 Post- 39.0	Pre-3.2
DHT, pg/mL (143-842)	Pre-40 Post-43	Pre-NA Post-NA	Pre-NA Post-NA	Pre-30 Post-38	Pre-40 Post-70	Pre-21 Post-24	NA
T/DHT	NA	NA	NA	NA	NA	>17	NA
Androstenedione, ng/mL, 0.05-0.35	<0.3	0.02	0.2	0.05	NA	0.05	NA
DHEA, ug/dL (M-133-440.7 F-30-335)	0.01	30.1	40.2	43	31	40	NA
T/A ratio (>0.8)	NA	NA	NA	0.5	0.5	NA	NA
AMH, ng/mL (55-210)	100.24	12	10	26.5	63	180	NA
Estradiol, pg/mL (M 15-71, F 57-227)	NA	2.3	0.01	NA	NA	NA	NA

**Table 4:** Radiological, genetic, and cytogenetics profile of genetically diagnosed DSD cases AR: autosomal recessive; AD: autosomal dominant; 5ARD2: 5-alpha-reductase type 2 deficiency; b/l: bilateral; CAH: congenital adrenal hyperplasia; CKD: chronic kidney disease; DHT: dihydrotestosterone; DHEA: dehydroepiandrosterone; DSD: differences of sex development; FISH: fluorescence in situ hybridization; FSH: follicle-stimulating hormone; G: gonads; GH: growth hormone; Hb: hemoglobin; hCG: human chorionic gonadotropin; HRT: hormone replacement therapy; K: potassium; L: left; LH: luteinizing hormone; LP: likely pathogenic; MGD: mixed gonadal dysgenesis; MLPA: multiplex ligation-dependent probe amplification; MS: Müllerian structures; N: normal; NA: not applicable; Na: sodium; NS: nephrotic syndrome; NV: not visualized; 17-OHP: 17-hydroxyprogesterone; OMIM: Online Mendelian Inheritance in Man; Plt: platelet count; RBS: random blood sugar; R: right; T: testosterone; TH: thyroid hormone; WES: whole-exome sequencing

S. No	Case 1	Case 2	Case 3	Case 4	Case 5	Case 6	Case 7	
Karyotype	46, XX	46, XX	46, XX	45,XO/47,XYY	46, XY	46, XY	46, XX	
FISH	SRY absent	SRY absent	SRY absent	SRY present	SRY present	SRY present	SRY absent	
USG pelvis	G: in scrotum; MS: absent	G: b/l ovary; MS: present	G: b/l ovary; MS: present	G: right testis-normal, left testis: NV; MS: absent	G-b/l testis in inguinal region; MS: absent	G: b/l testis present inguinal; MS-absent	G: b/l ovary; MS: present	
MRI abdomen	Testes-b/l in scrotum, MS: absent	Normal	NA	Right testis normal, Left testis: streak; Bifid right kidney MS-rudimentary	b/l testis in inguinal region, MS: absent, kidney: normal	G: b/l testis inguinal, MS: absent, blind vaginal pouch	NA	
MRI brain	Normal	NA	NA	normal	normal	NA	NA	
Gonadal biopsy	b/l testicular tissue present	NA	NA	Left gonad: streak ovaries and fallopian tubes	NA	NA	NA	
WES/MLPA								
Gene	RSPO-1	CYP21A2	CYP19A1	NA	WT1	SRD5A2		CYP21A2
Location	Exon 4	Intron 2	Exon 10	NA	Exon 7	Exon 5		
Variant	c.254del (p.Asp85AlafsTer7)	c.293-13C>G			c.1193G>C(p.Cys398Ser)	c.737G>A(p.R246Q)		NM_000500.7:c.293-13C/A>G
Zygosity	Homozygous	Homozygous	Homozygous	NA	Heterozygous	Homozygous		Homozygous
Disease (OMIM)	Palmoplantar hyperkeratosis and true hermaphroditism or Palmoplantar hyperkeratosis with squamous cell carcinoma of skin and sex reversal (OMIM#610644)	Congenital adrenal hyperplasia, due to 21-hydroxylase deficiency	Aromatase deficiency	NA	Denys-Drash syndrome; Fraser syndrome; Nephrotic syndrome type 4	5 alpha reductase type 2 deficiency		Congenital adrenal hyperplasia, due to 21-hydroxylase deficiency
Inheritence	AR	AR	AR	NA	AD	AR		AR
Classification	LP	LP	LP	NA	LP	LP		LP
Final diagnosis	46 XX testicular DSD	CAH	Aromatase deficiency	MGD	Denys-drash syndrome	5ARD2 deficiency		CAH
Interventions	Urethroplasty	HRT	HRT	Left gonadectomy and urethroplasty	Urethroplasty and orchidopexy	HRT, DHT cream		HRT

Case 2

One-month-old infant, firstborn of non-consanguineous marriage, reared as a girl, presented with atypical genitalia, vomiting, features of shock and hypoglycemia. Genital examination revealed clitoromegaly, hyperpigmentation with normal urethral and vaginal opening (Figure 2b and Table 2). Investigations revealed a 46, XX karyotype, hyponatremia, hyperkalemia, hypoglycemia (random blood sugar 30 mg/dL), and high 17-OHP. Detailed laboratory and radiological investigations are given in Tables 3-4. WES identified a pathogenic variant in the CYP21A2 gene (c.293-13C>G, intron 2) in a homozygous state (Table 4). MLPA demonstrated a probe ratio of 0 for the c.293-13C>G variant, confirming complete loss of the corresponding target region. Additionally, a heterozygous deletion spanning the 5′UTR to exon 7 was identified, with probe ratios of 0.50-0.51, consistent with a single-copy deletion of the affected segment. She was diagnosed with CAH due to 21-alpha hydroxylase deficiency and was started on lifelong hormone replacement therapy with hydrocortisone and fludrocortisone.

Case 3

Our next patient was a 12-year-old developmentally normal child, the second born of a third-degree consanguineous marriage, who presented with progressive clitoromegaly. She was born with atypical genitalia (clitoromegaly), but her parents did not seek medical advice at that time due to social stigma. She was reared as a girl. Genital examination revealed clitoral length of about 2.5 cm with normal urethral and vaginal openings (Figures 2c-2d and Table 2). Karyotype was 46 XX. Biochemical parameters revealed normal 17-OHP, elevated testosterone and DHT, and very low estradiol levels (Table 3). WES reported a homozygous nonsense variant in exon 10 of the CYP19A1 gene, a pathogenic variant associated with aromatase deficiency. She was started on estrogen replacement therapy and was advised to undergo appropriate surgical intervention.

Case 4

Our next patient was a developmentally normal 10.5-year-old child reared as a boy, firstborn of a non-consanguineous marriage, who presented with micropenis, asymmetric and small scrotum, penoscrotal hypospadias and unilateral cryptorchidism (Figure 2e and Table 2). He also had primary hypothyroidism. Biochemical parameters revealed low testosterone and DHT levels, both pre- and post-hCG stimulation (Table 3). MRI of the abdomen revealed a streaked left testis, a normal right testis, rudimentary Müllerian-like structures, and a bifid right kidney (Table 4). Karyotype showed an abnormal finding with mosaicism: 45, XO/47, XYY. 45, XO was seen in 76% of the cells and 47, XYY in 24% of the cells (Figures 3a-3b). FISH confirmed the karyotype finding and the presence of the SRY gene (orange signals) on the Y chromosome (Figure 3c). He underwent left gonadectomy and urethroplasty. Histopathology of the left gonad showed streak ovaries and fallopian tubes. He was diagnosed with MGD. Parents were counselled regarding the rearing of this child and hormone replacement therapy.

Case 5

A six-year-old developmentally normal child reared as a boy presented with microphallus, small and bifid scrotum, bilateral cryptorchidism and penoscrotal hypospadias (Figure 2f). He also had focal segmental glomerulosclerosis (FSGS) from the age of two years, for which he was on on-off prednisolone and tacrolimus for the last three years. His father also had chronic kidney disease (CKD), for which he underwent a kidney transplant at 24 years of age. Post-transplant, he was doing well. A genetic test for the father was not performed due to financial constraints. Detailed demographic and clinical findings are presented in Table 2. His karyotype was 46 XY. Biochemical evaluation showed low testosterone and dihydrotestosterone (DHT) levels (Table 3). MRI of the abdomen identified bilateral testes in the inguinal region and absent Müllerian structures (Table 4). The WES reported a heterozygous missense variation in exon 7 of the WT1 gene (chr11:g.32396328C>G; depth: 91x) (Table 4). He was diagnosed with Denys-Drash syndrome (DDS) and was put on surveillance for malignancy, especially Wilms’ tumour.

Case 6

A nine-month-old developmentally normal infant, firstborn of a third-degree consanguineous marriage, reared as a girl, presented with microphallus, bifid scrotum, penoscrotal hypospadias, a blind vaginal opening and bilateral palpable gonads in the inguinal region (Figures 2g-2h and Table 2). The karyotype was 46 XY. Testosterone was high, and DHT was low both pre- and post-HCG stimulation (Table 3). T/DHT ratio was 18.7 (>10). AMH was normal. MRI abdomen identified a blind vaginal pouch and absence of Müllerian structures (Table 4). WES reported a homozygous missense variant c.737G>A (p.R246Q) in exon 5 of the SRD5A2 gene (Table 4). The infant was diagnosed with 5-alpha reductase type 2 deficiency. Parents were counselled regarding the rearing of this child, hormone replacement therapy and appropriate surgical interventions. Parents opted for male rearing.

Case 7

A one-month-five-day-old infant, firstborn of a non-consanguineous marriage with an uneventful antenatal and natal period, reared as a girl, presented with atypical genitalia, vomiting, features of shock and hypoglycemia. Genital examination revealed clitoromegaly, hyperpigmentation with normal urethral and vaginal opening (Table 2). Investigations revealed a 46, XX karyotype, hyponatremia, hyperkalemia, hypoglycemia (random blood sugar 40 mg/dL), and high 17-OHP. Detailed laboratory and radiological investigations are given in Tables 3-4. WES reported a homozygous pathogenic variant NM_000500.7:c.293-13C/A>G(12G) in the CYP21A2 gene (Table 4). She was diagnosed with CAH due to 21-alpha hydroxylase deficiency and was started on lifelong hormone replacement therapy with hydrocortisone and fludrocortisone.

## Discussion

Management of DSD involves a multidisciplinary team from different specialities [10]. In our country, where consanguinity is found in significant numbers, the clinical utility of molecular genetics is unequivocally clear. Genetic tools such as microarray and NGS have modified the traditional approach, which was more stratified and stepwise, into a more integrative form for diagnosing and managing DSD. These tests can detect genetic variations known to be associated with DSDs, discover novel genetic variants, and elucidate novel mechanisms of gene regulation.

In the present study, the age range of our patients was one month to 14 years, and most presented within 10 years of age, which was similar to other Indian studies [11-13]. The most common DSD in our cohort was 46, XY DSD (60%), followed by 46, XX DSD (30%) and sex chromosomal DSD (10%), consistent with findings of Jahagirdar et al. and Walia et al. [11,13]. In 46, XX DSD, CAH was the most common diagnosis; in 46, XY DSD, AIS was the most common, followed by 5-alpha-reductase type 2 deficiency. These findings were consistent with other Indian and Western studies [11-14]. Of 20 DSD patients, we obtained genetic confirmation of diagnosis in seven cases. So, a diagnostic yield of 35% was observed in the present study, which was similar to that of Dong et al. (38%) and Fan et al. (28%) from China [14,15]. Eggers et al., in one of the world’s largest international cohorts of DSD patients, reported diagnostic yields of 43% for 46, XY DSD and 19% for 46, XX DSD with genetic testing [16]. A few Indian studies have examined the genetic aetiology of DSD in children [17-20]. The genetic aetiology of DSD in the present study was associated with the RSPO1, CYP21A2, CYP19A1, WT1, and SRD5A2 genes.

RSPO1

RSPO1 is an activator of the WNT signalling pathway and is a key factor for ovarian differentiation. It has a family of four secretory proteins, RSPO1-4. Germline mutations in RSPO1 and RSPO4 are associated with developmental disorders of the reproductive organs and nails, respectively. There are very few reported cases of RSPO1-associated DSD [21,22]. It is also associated with squamous cell carcinoma of palmoplantar skin. The child (Case 1) already had palmoplantar hyperkeratosis in both of his palms. It predisposes him to future risk of squamous cell carcinoma. Parents were counselled about the future risk of malignancy.

CYP21A2

The 21-hydroxylase active genes CYP21A2, CYP21 or CYP21B are located on chromosome 6p21.3. A mutation in the CYP21A2 gene leads to congenital deficiency of the 21-alpha-hydroxylase enzyme, an important rate-limiting enzyme in the steroidogenic pathway. The variant c.293-13C/A>G (12G) is the most common variant found in CAH patients in India. It is one of the most common causes of 46 XX DSD [11-13]. It can present as salt-wasting, non-classic, or simple virilising. Both our patients (Cases 1 and 7) presented in adrenal crisis.

CYP19A1

This gene encodes the human aromatase enzyme, part of the CYP450 superfamily. It converts androgens to estrogens in gonadal and peripheral tissues. More than 30 mutations have been identified in the CYP19A1 gene. The majority of these are located in exons 9 and 10 [23]. Our patient had a mutation in exon 10 of the CYP19A1 gene. It’s a very rare disorder.

45,X/47,XYY

This kind of chromosomal mosaicism is very rare. It has an incidence of 1.7/10,000 pregnancies [24]. Very few cases have been reported in the literature [24,25]. It results from postzygotic mitotic non-disjunction. Most of them are raised males, likewise in our case (Case 4). The gonads range from dysgenetic testes and streak ovaries to ovotestis. Our patient had streak ovaries on the histopathological findings of left gonadectomy. In the Farrugia et al. [24] study, 48% of patients had other associated anomalies, mostly cardiac and renal; likewise, our patient (Case 4) had a bifid right kidney. The future fertility potential of mosaic children is unclear in the literature.

WT1

WT1, a zinc-finger transcription factor, is located on chromosome 11p13. Abnormalities of WT1 are associated with a large spectrum of disorders, including Wilm’s tumour, glomerulopathy, FSGS, congenital anomalies of the kidney and urinary tract (CAKUT), DSD and gonadoblastoma [5,14]. The 46XY DSD that arises in these syndromes are due to a disorder of gonadal development, mainly partial gonadal dysgenesis. DDS, Frasier syndrome, and Wilms tumour-aniridia-genitourinary anomalies-intellectual disability (WAGR) are common WT1-associated syndromes. Germline WT1 mutations predominantly located on exons 7, 8 or 9 are frequently seen in DDS, while mutations predominantly located in intron 9 are frequently seen in Frasier syndrome [5]. In our patient, it was observed in exon 7. He was diagnosed with DDS. The parents were counselled about the future risk of Wilms' tumour, and the child was put on surveillance for the same.

SRD5A2

The SRD5A2 gene, present on chromosome 2 (2p23), consists of 5 exons and 4 introns. A mutation in an exon is most often associated with 5-alpha reductase deficiency. It is an autosomal recessive condition caused by homozygous or compound heterozygous variants in the SRD5A2 gene [20]. Consanguinity is present at a higher frequency in this condition; our patient (Case 6) was also the product of a third-degree consanguineous marriage. Gender dysphoria is as high as more than 50% in females with 5-alpha reductase type 2 deficiency [26]. Our patient (Case 6) was reared as female, but after the confirmed genetic diagnosis, the parents opted for male gender rearing.

No study comes without limitations. Our study was limited by its retrospective nature, small patient number, lack of parental segregation data, limited long-term outcome data, and single-centre basis. The strength was that we had a complete clinical, biochemical, radiological, histopathological, genetic and cytogenetic workup of these children. The present study showed a diagnostic yield of almost 35% in genetic testing, which could open new avenues. Because of the upfront genetic testing in our two patients (Cases 1 and 5), the future risk of malignancy was identified in them, and they were put on surveillance.

## Conclusions

DSDs are an evolving entity. Parents should be counselled to seek early medical advice and not to get stigmatised by it. In the present study, 46, XY DSD was more common than 46, XX. Among 46, XX DSD, CAH was the most common aetiology, while AIS was the most common aetiology in 46, XY DSD. The present study highlights the importance of upfront molecular genetic testing (diagnostic yield of 35%) in DSD patients, which can predict the natural course of the disease, potential for fertility, gender rearing, future risk of malignancy, and recurrence in subsequent pregnancies. Some rare genes, like RSPO1, were identified through genetic testing. Molecular testing is a valuable adjunct to clinical, biochemical, radiological, and histopathological evaluation rather than a standalone diagnostic tool. Accurate diagnosis and optimal management of DSD require a multidisciplinary team approach.
